# Prognostic Value and Implication for Chemotherapy Treatment of ABCB1 in Epithelial Ovarian Cancer: A Meta-Analysis

**DOI:** 10.1371/journal.pone.0166058

**Published:** 2016-11-03

**Authors:** Si Sun, Jing Cai, Qiang Yang, Yapei Zhu, Simei Zhao, Zehua Wang

**Affiliations:** Department of Obstetrics and Gynecology, Union Hospital, Tongji Medical College, Huazhong University of Science and Technology, Wuhan, Hubei, People’s Republic of China; University of Nebraska Medical Center, UNITED STATES

## Abstract

**Background:**

Chemotherapy resistance is reported to correlate with up-regulation of anti-tumor agent transporter ABCB1 (p-gp) in epithelial ovarian cancer (EOC), but the results remain controversial. To reconcile the results, a systematic review followed by meta-analysis was performed to assess the association between high ABCB1 status or *ABCB1* gene variants and overall survival (OS), progression free survival (PFS), and total response rate (TR) in patients with EOC.

**Materials and Methods:**

Electronic searches were performed using Pubmed, EMBASE, Web of Science and Chinese Wanfang databases from January 1990 to February 2016. Summary hazard ratio (HR), risk ratio (RR) and 95% confidence intervals (CIs) were combined using fixed or random-effects models as appropriate.

**Results:**

Thirty-eight retrospective studies of 8607 cases qualified for meta-analysis were identified. Our results suggested that ABCB1 over-expression was significantly associated with unfavorable OS (HR = 1.54; 95% CI, 1.25–1.90), PFS (HR = 1.49; 95% CI, 1.22–1.82) and TR (RR = 0.63; 95% CI, 0.54–0.75). After adjustment for age, clinical stage, residual disease, histological type and tumor grade, high ABCB1 status remained to be a significant risk factor for adverse OS and PFS. Patients with recurrent ABCB1 positivity suffered from poorer OS than those with primary ABCB1 positivity. However, stratified by chemotherapy regimen, inverse correlation between high ABCB1 status and poor OS, PFS and TR were only found in patients underwent platinum-based chemotherapy but not in patients received standard platinum/paclitaxel-based chemotherapy. No evidence was found for any association between *ABCB1* gene polymorphisms and OS, PFS or TR.

**Conclusion:**

High ABCB1 status is significantly associated with chemo-resistance and poor prognosis in patients with EOC. Large-scale, prospective studies are needed to assess the clinical value of ABCB1 expression in EOC more accurately.

## Introduction

Ovarian cancer is the most lethal gynecological malignancy worldwide [1]. Epithelial ovarian cancer (EOC) accounts for over 90% of all ovarian cancers [2]. As its progression is usually asymptomatic and unpredictable, most (approximately 85%) patients were diagnosed at late stage, in whom cytoreductive surgery combined with platinum-based chemotherapy represents the current standard procedure [3]. Six cycles of taxane plus platinum chemotherapy represents the most widely used regimen for EOC; other platinum-based regimens included PAC (platinum + doxorubicin + cyclophosphamide) and PC (platinum + cyclophosphamide). Although this treatment procedure can achieve an overall response rate higher than 70% in patients with ovarian cancer, a great proportion (approximately 75%) of them suffer a recurrence within 2 to 3 years after initial treatment, which leads to poor outcomes [4]. The high recurrence rate is associated with the development of acquired chemo-resistance. Repeated chemotherapeutic stimulation induces biological changes in tumor cells allowing them to survive under chemotherapy. Identification of prognostic biomarkers predicting chemotherapy response was essentially necessary for individual therapy.

Insufficient accumulation of anti-cancer agents in tumor cells resulting from increased efflux and/or decreased influx is a critical mechanism of chemo-resistance [5,6]. ABCB1 (ATP binding cassette B1, also known as multidrug resistance protein 1, MDR1 or p-glycoprotein, p-gp) is a cellular surface protein, which transports a variety of chemotherapy drugs such as paclitaxel, docetaxel, doxorubicin and topotecan and plays a major role in cellular detoxification [7–10]. Functional experiments revealed that up-regulated ABCB1 expression impaired the sensitivity to cisplatin, paclitaxel, docetaxel and doxorubicin in ovarian cancer cell lines [11]. A substantial literature interrogated the relationship between ABCB1 status (expression or polymorphisms) and clinical outcomes such as overall survival (OS), progression free survival (PFS) and total response (including complete response and partial response according to Response Evaluation Criteria In Solid Tumors) to chemotherapy (TR) in EOC patients [12–55]. Although a great number of studies evaluated the effect of ABCB1 on EOC patients, the association between ABCB1 and EOC prognosis was still under debate. While certain amount of studies reported that high ABCB1 could predict unfavorable prognosis [23,28,30,31,34,35], quite a few did not find any significant association between ABCB1 and survival in patients with EOC [18,27,32]. The possible reasons for these inconsistent results might include (i) heterogeneities of the basic characteristics of the studies such as sample sizes, EOC subtypes, clinical stage, and populations, (ii) different intervention such as residual disease after surgery, time point of ABCB1 measurement and (iii) different methodology such as detection methods and procedures. Therefore, we performed a systematic and comprehensive meta-analysis to assess more precisely the correlation between ABCB1 status and EOC prognosis based on individual studies.

## Methods

This meta-analysis was performed according to the PRISMA-statement for systematic review and meta-analysis of genetic association studies (S1 and S2 Checklists).

### Search strategy

Thorough electronic search of Pubmed, Embase, Web of Science and Chinese Wanfang databases from January 1990 to Feb 2016 was conducted with the following key words: ‘ovarian cancer’, ‘ovarian carcinoma’ or ‘ovarian neoplasm’ and ‘ABCB1’ or ‘p-gp’. The search strategy was complemented by reviewing the similar articles and by examining the references of the retrieved literature. Additionally we examined the authors of each study to avoid overlap of cases. Only the most recent study was included when overlap occurred between studies.

### Publication Selection and Quality Assessment

Two authors reviewed the studies independently to decide which were eligible for inclusion. The included studies had to meet the following criteria: 1) focused on epithelial ovarian carcinoma and evaluated correlation between ABCB1 status and prognosis of the patients; 2) published as full text in English or in Chinese; 3) analyzed ABCB1 status with practical and comparable method; 4) abided by the Hardy Weinberg Equilibrium (HWE) when performing gene associated analysis. The primary outcome was OS and the secondary outcome included PFS and TR. The methodology of each study was assessed independently by two authors according to the Newcastle-Ottawa Scale (NOS) for assessing the quality of nonrandomized studies in meta-analysis. Each study was scored according to three aspects: selection, comparability and exposure. The maximum score of each aspect was 4, 2 and 3 respectively, adding up to a total score of 9. Higher score stood for better quality (S1 File).

### Data Extraction and Statistical Methods

The data extracted from the included studies were authors, year of publication, countries, sizes of population, histology, clinical stage, follow-up time, treatment, tissue type, point of time when transporters are measured, assay method and corresponding scoring system, the results of survival analysis, the number of cases and controls, and response to chemotherapy. Two authors reviewed the literatures and extracted the data independently. If disagreement occurred, a third author was conferred with thus the issue be discussed altogether.

For time to event survival analysis, we assessed the effect of ABCB1 status on prognosis by hazard ratio (HR). For each study, the HR and its 95% confidence interval (CI) were retrieved. If these parameters were not available in studies, we used the software GetDATA Graph Digitizer 2.25 to extract the specific survival rates according to the Kaplan-Meier curves to calculate the HR by the methods described by Tierney et al [56]. For dichotomous outcomes such as TR, we measured the impact of ABCB1 status on the prognosis by risk ratio (RR). For each study, we extracted the number of patients who experienced the events of interest and the number of patients who were free from the events at the end point to calculate the RR and its 95% CI.

Meta-analysis was performed for OS, PFS and treatment response. For each endpoint, the analyses were stratified by ABCB1 detection methods, histology, clinical stage, geographical region of studies and chemotherapy regimens. Generally the individual HR and RR were pooled according to the method reported by DerSimonian, R et al. and Mantel, N et al. Fixed inverse variance model and random model were used to calculate pooled HRs and RRs of studies with class I-II heterogeneity (I^2^ <50%) and those with class III-IV hererogeneity (I^2^ ≥50%), respectively [57,58]. The heterogeneity between studies was assessed by I^2^ and p-value according to the method described by Higgins et al [59]. The effect of ABCB1 on prognosis was considered significant only when the 95% CI for HR and RR did not overlap with 1. Visual inspection of symmetry of funnel plots and Egger’s test were used to assess publication bias [60]. A two-sided *P* value < 0.05 was considered of statistical significance. All analyses were carried out using STATA14 (MP-Parallel Edition, College Station, Texas 77845 USA).

## Results

### Study characteristics

**Fig 1:**
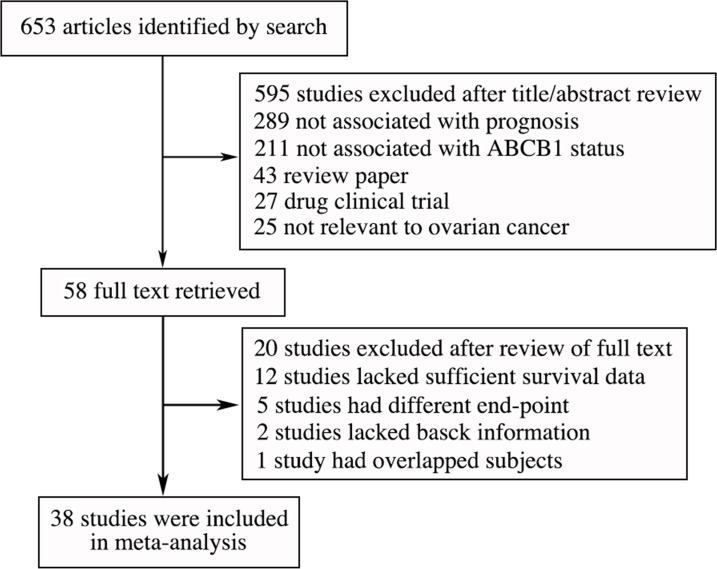
Flow chart of literature search and selection.

The procedure of study selection was presented in a flowchart (Fig 1). Initially, 653 articles related to the keywords were identified after removal of duplicates. Among these articles, 595 were excluded by review of title and abstract as they did not report on ABCB1, published as reviews or focused on ABCB1 cytological mechanisms and function. Full texts were retrieved of the remaining 58 studies and 20 of them were excluded after review of full text (S1 Table). Finally, 38 eligible studies involving 8607 cases were identified, including 23 studies for ABCB1 protein (p-gp) expression (Table 1), 9 for ABCB1 mRNA expression, and 7 for *ABCB1* SNPs (Table 2).

**Table 1:** Characteristics of studies that identify ABCB1 protein (p-gp) expression in epithelial ovarian cancer.

Detection method	First Author	Year	Country	No. of Cases	Stage	Histology	Anti-body	Score	Cut-off point	Tissue Type	Time point	Chemotherapy	End-point
IHC	Matsuo	2014	USA	112	I-IV	s	JSB1	standard	5%	FFPE	1	2	OS, PFS
	**Lu D**	2011	China	80	I-IV	s,m,e	ZSGB	standard	10%	FFPE	1	1,2	OS
	**O'Tool**	2007	Ireland	77	I-IV	s,m,c	JSB1	standard	10%	FFPE	1	1,2	OS, PFS
	Oda	2007	Japan	53	I-IV	s,e	JSB1	standard	10%	FFPE	1	2	OS, PFS
	Wei	2007	China	42	III	all	C219	standard	20%	FFPE	1	2	TR
	**Yakirevich**	2006	USA	60	I-IV	s	JSB1	standard	10%	FFPE	1	2	OS, TR
	**Surowiak**	2006	Poland	43	III-IV	all	C219	standard	20%	FFPE	1,2	1	OS, PFS, TR
	Obata	2006	Japan	60	III-IV	s,e	C-19	standard	5%	FFPE	1	1,2	TR
	**Goto**	2006	Japan	21	IIIC~IV	s	JSB1	standard	20%	FFPE	1	2	TR
	**Raspollini**	2005	Italy	52	III	s	MDR8	standard	30%	FFPE	1	1	TR
	**Penson**	2004	USA	32	II~IV	s,e,c	C219, c494	standard	20%	FFPE	1,2	2	OS, TR
	**Ikeda**	2003	Japan	93	II~IV	all	C-19	standard	10%	FFPE	1	1,2	OS
	**Raspollini**	2003	Italy	83	III	s	MDR8	standard	30%	FFPE	1	1	OS, PFS, TR
	**Raspollini**	2002	Italy	22	III	s,e	MDR8	standard	30%	FFPE	1	1	PFS, TR
	Itamochi	2002	Japan	131	III~IV	s,c	C494, C-20	standard	10%	FFPE	1	1	TR
	**Zhang**	2001	China	27	I-IV	all	C219	standard	10%	FFPE	2	1	TR
	**Baekelandt**	2000	Norway	73	III	all	JSB1	standard	10%	FFPE	1	1	OS, PFS, TR
	Yokoyama	1999	Japan	22	I-IV	all	LSAB	standard	30%	FFPE	1	1	PFS, TR
	Arts	1999	Netherlands	112	I-IV	all	JSB1	standard	10%	FFPE	1	1,2	PFS, TR
	**Khalifa**	1997	Canada	75	I-IV	all	JSB1	standard	10%	FFPE	1,2	1	OS
	Van	1995	Netherlands	89	I-IV	all	JSB1	standard	5%	FFPE	1	1	PFS,TR
	Izquierdo	1995	Netherlands	57	III-IV	s,m,e	JSB1	standard	10%	FFPE	1	1	TR
WB	Joncourt	1998	Switzerland	39	I-IV	all	Customized*	-	positive	Frozen	1	1	TR

IHC: Immunohistochemistry; WB: Western blot

First Author: bolded when the result of ABCB1 association was significant

Histology: all = serous, mucinous, endometrial and clear cell carcinoma with or without mixed histology, s = serous ovarian cancer, m = mucinous ovarian cancer, e = endometrial ovarian cancer, c = clear cell ovarian cancer

Score: standard = score procedures were performed double-blindly by at least one pathologist according to standard scoring systems

Tissue Type: FFPE = formalin-fixed and paraffin-embedded

Time point: the time point of specimen obtained, 1 = specimens obtained during primary surgery, 2 = specimens obtained from recurrent tumor

Treatment: 1 = platinum-based chemotherapy, 2 taxane-containing chemotherapy

*The antibody used was a polyclonal rabbit antiserum raised against amino acids 1205–1224 of the human mdr protein. This peptide (ALDTESEKV- VQEALDKAREG) was made by Multiple Peptide Systems, Inc. (San Diego, CA).

**Table 2:** Characteristics of studies that identify ABCB1 mRNA expression and *ABCB1* gene variants in epithelial ovarian cancer.

	First Author	Year	Country	No. of Cases	Stage	Histology	Detection method	Tissue Type	Time point	Chemotherapy	End-point
ABCB1 mRNA expression	Johnatty	2013	Australia	143	I-IV	s	PCR	frozen	1	2	OS, PFS
	**Lu L**	2007	USA	206	I-IV	all	PCR	frozen	1	1,2	OS, PFS
	**Materna**	2004	Germany	61	I-IV	all	PCR	frozen	1	1,2	OS, PFS
	Nakayama	2002	Japan	82	I-IV	all	PCR	frozen	1	1,2	OS
	Galani	2002	Greece	22	III-IV	all	PCR	frozen	1	1,2	TR
	Kamazawa	2002	Japan	27	III-IV	s,e,c	PCR	frozen	1	2	TR
	**Wang**	1998	China	27	I-IV	all	PCR	frozen	1	1	TR
	**Zhu L**	1997	China	32	III	s,e,c	PCR	frozen	1	1	TR
	**Kavallaris**	1996	Australia	53	I-IV	all	PCR	frozen	1	1	PFS
*ABCB1* gene variants	Tecza K	2015	Poland	126	I-IV	all	ASA-PCR, multiplex-PCR,RFLP-PCR	blood	1	2	PFS
	Johnatty	2013	Australia	4450	I-IV	all	Illumina Infinium iSelect BeadChip	blood	1	2	OS, PFS
	Tian	2012	USA	511	III-IV	all	Sequenom iPLEXTMGOLD Aassay and MALDI-TOF	blood	1	2	OS, PFS
	Prema	2011	USA	445	I-IV	all	Illumina Veracode Assay	blood	1, 2	1	PFS
	Bergmann	2011	Denmark	119	II-IV	all	Pyrosequencing and TaqMAN® predesigned SNP genotyping assays	FFPE	1	1	OS
	Grimm	2010	Austria	106	I-IV	all	Pyrosequencing	blood	1	2	OS, PFS
	Johnatty	2008	Australia	309	I-IV	all	MALDI-TOF	blood	1	2	OS, PFS

First Author: bolded when the result of ABCB1 association was significant

Histology: all = serous, mucinous, endometrial and clear cell carcinoma with or without mixed histology, s = serous ovarian cancer, m = mucinous ovarian cancer, e = endometrial ovarian cancer, c = clear cell ovarian cancer

Tissue Type: FFPE = formalin-fixed and paraffin-embedded

Time point: the time point of specimen obtained, 1 = specimens obtained during primary surgery, 2 = specimens obtained from recurrent tumor

Treatment: 1 = platinum-based chemotherapy, 2 taxane-containing chemotherapy.

The number of studies addressing OS, PFS, and TR was 19, 19, and 21 respectively. In addition to the basic characteristics, 6 and 4 studies assessed the impact of ABCB1 on OS [20,23,29,37,45,48] and PFS [20,29,37,45], respectively, adjusted for age, clinical stage, residual disease, histological type and tumor grade by multivariate analysis (Tables 1 and 2). For p-gp intensity, IHC results were appraised by measuring the intensity of reaction and proportion of positive cells. For measurement of *ABCB1* gene expression by PCR, most of the studies (8/9) used the median as the cut-point according to the distribution of each cohort. The distributions of ABCB1 genotype frequencies in all 7 studies did not depart from Hardy-Weinberg equilibrium. Most of the studies revealed no correlation between common *ABCB1* variants and poor prognosis (6/7).

### Meta-analysis of relationship between ABCB1 status and OS in EOC

**Fig 2:**
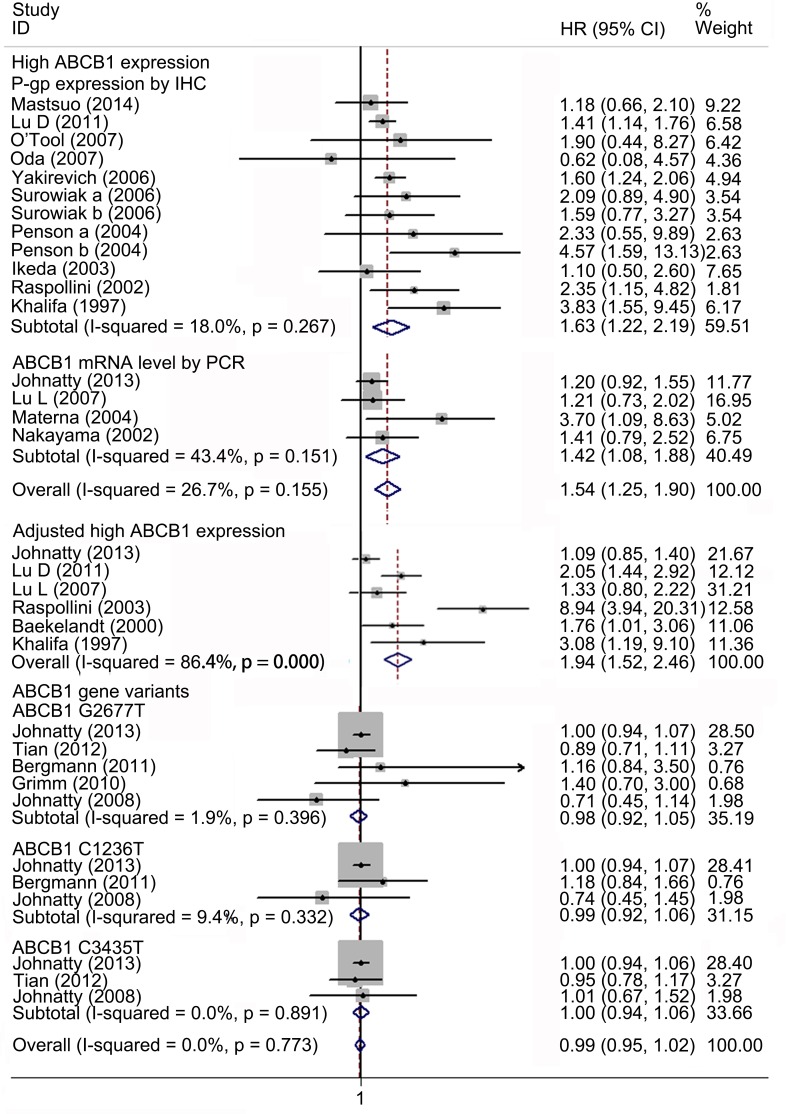
Forest plots presenting HRs of EOC OS for high ABCB1 expression, adjusted results and *ABCB1* gene variants. HR = hazard ratio; EOC = epithelial ovarian cancer; OS = overall survival.

**Fig 3:**
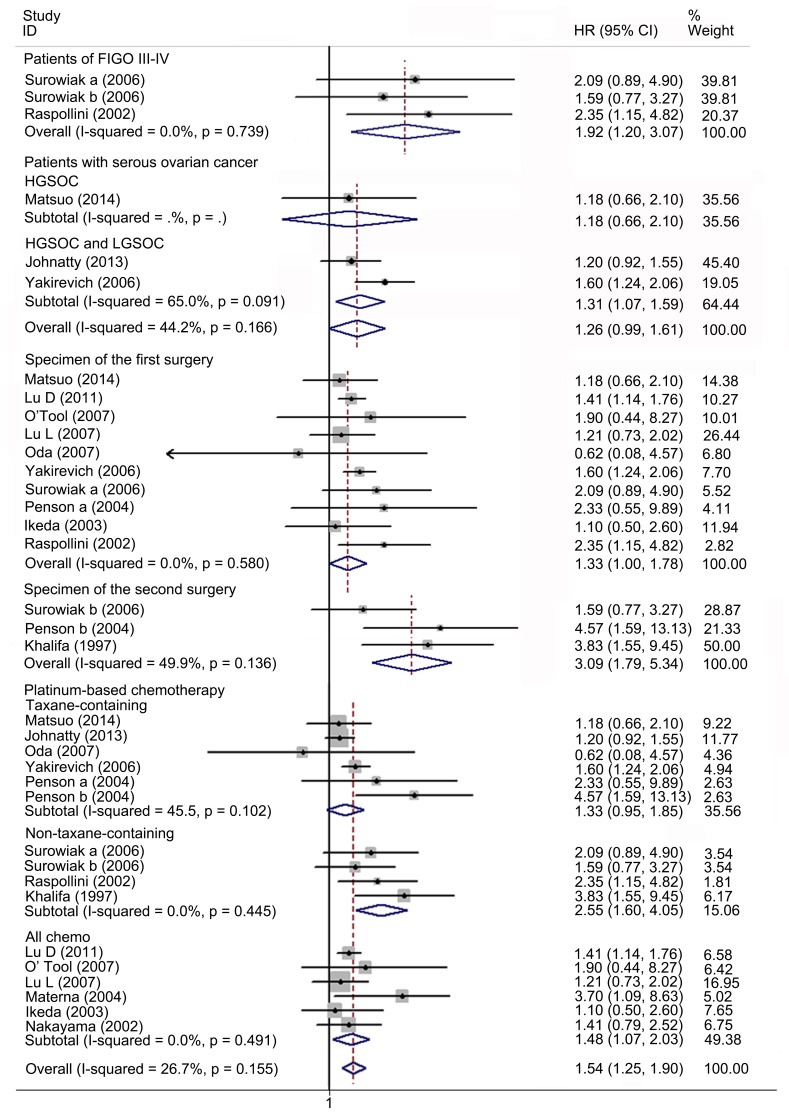
Forest plots of subgroup meta-analysis presenting HRs of EOC OS for high ABCB1 expression. HR = hazard ratio; EOC = epithelial ovarian cancer; OS = overall survival.

**Fig 4:**
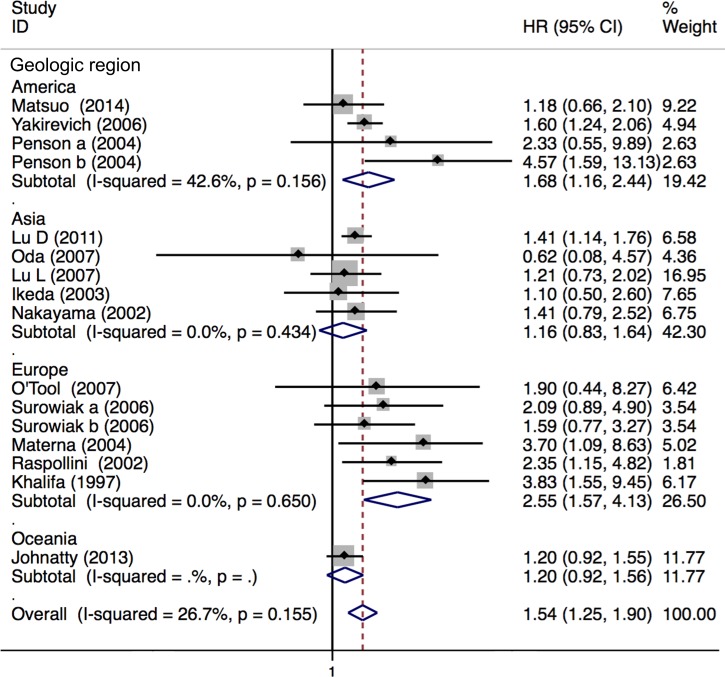
Forest plots of HRs of EOC OS for high ABCB1 expression stratified by geographical region. HR = hazard ratio; EOC = epithelial ovarian cancer; OS = overall survival.

Nineteen studies with 6635 patients were included in the meta-analysis of OS. Fifteen studies with 1140 patients reported the association between high ABCB1 expression and OS. Overall, high ABCB1 expression was correlated with a poor OS (HR, 1.54; 95% CI: 1.25–1.90) in EOC. The effect of ABCB1 expression on OS remained significant in the IHC (HR, 1.63; 95% CI: 1.22–2.19), the PCR (HR, 1.42; 95% CI: 1.08–1.88), the FIGO III-IV (HR, 1.92; 95% CI: 1.20–3.07), the ABCB1 expression in recurrent tumor (HR, 3.09; 95% CI: 1.79–5.34), the platinum-based non-taxane-containing chemotherapy (HR, 2.55; 95% CI: 1.60–4.05), the America (HR, 1.68; 95% CI: 1.16–2.44) and the Europe (HR, 2.55; 95% CI: 1.57–4.13) subgroups. Similarly, when adjusted for residual disease (N = 6), age (N = 4), FIGO stage (N = 4), histological type (N = 2) and tumor grade (N = 1), high ABCB1 expression remained significantly associated with OS (HR, 1.94; 95% CI: 1.52–2.46). However, an inverse correlation was not found in the serous tumor, in the Asia, in the Oceania, in the patients with primary tumors, nor in the taxane-containing chemotherapy subgroups. We found that in the serous subgroup, the correlation between ABCB1 expression and OS became significant after excluding study specifically identifying high-grade serous ovarian cancer (HGSOC) (HR, 1.31; 95% CI: 1.07–1.59). For *ABCB1* gene polymorphisms, although rs2235023, rs13237132, rs12334183, rs10264990, and rs4148732 were previously reported to be associated with ovarian cancer outcome, evidences from a study of more than 10000 cases suggested these candidate polymorphisms were not correlated with EOC prognosis [19]. Therefore we only evaluated the impact of rs2032582, rs1128503 and rs1045642 on prognosis. Five studies with 5495 patients reported association between these polymorphisms and OS in EOC [20,21,24,25,54]. No evidence of correlation between *ABCB1* gene SNPs and OS was found (HR, 0.99; 95% CI: 0.95–1.02). The heterogeneity between studies in these analyses was acceptable (Figs 2–4 and Table 3).

**Table 3:** Pooled HRs or RRs, heterogeneities and public cation biases of meta-analyses and subgroup analyses.

	No. of studies	HR (95%CI) or RR (95%CI)^a^	HR and RR*P* value	Heterogeneity*P* value	I^2^ value (%)	Bias *P* value
OS	19					
High ABCB1 expression	15	1.54 (1.25–1.90)	0.000	0.267	18.0	0.078
ABCB1 detected by IHC	11	1.63 (1.22–2.19)	0.000	0.151	43.4	0.206
ABCB1 detected by by PCR	4	1.42 (1.08–1.88)	0.019	0.155	26.7	0.171
FIGO III-IV	2	1.92 (1.20–3.07)	0.006	0.739	0.0	-
Serous tumor	3	1.26 (0.99–1.61)	0.061	0.166	44.2	0.766
ABCB1 expression in primary tumor	10	1.33 (1.00–1.78)	0.049	0.580	0.0	0.888
ABCB1 expression in recurrent tumor	3	3.09 (1.79–5.34)	0.000	0.136	49.9	0.182
Platinum-based chemotherapy	14	1.54 (1.25–1.90)	0.000	0.155	26.7	0.078
Taxane-containing chemotherapy^b^	5	1.33 (0.95–1.85)	0.093	0.102	45.5	0.613
Non-taxane-containing chemotherapy^c^	3	2.55 (1.60–4.05)	0.000	0.445	0.0	0.311
All chemotherapy^d^	6	1.48 (1.07–2.03)	0.017	0.491	0.0	0.505
Geographical region						
America	3	1.68 (1.16–2.44)	0.006	0.156	42.6	0.957
Asia	5	1.16 (0.83–1.64)	0.382	0.434	0.0	0.104
Europe	5	2.55 (1.57–4.13)	0.000	0.650	0.0	0.327
Oceania	1	1.20 (0.92–1.56)	0.171	-	-	-
High ABCB1 expression adjusted for residual disease	4	1.94 (1.52–2.46)	0.000	0.000	86.4	0.080
*ABCB1* gene variants	6	0.95 (0.99–1.02)	0.989	0.053	54.1	0.748
PFS	19					
High ABCB1 expression	12	1.49 (1.22–1.82)	0.000	0.000	73.6	0.146
ABCB1 detected by IHC	8	1.37 (1.05–1.79)	0.042	0.000	81.3	0.122
ABCB1 detected by by PCR	4	1.67 (1.24–2.26)	0.001	0.099	52.1	0.123
FIGO III-IV	3	1.97 (1.40–2.78)	0.000	0.000	88.0	0.068
Serous tumor	2	1.14 (0.82–1.59)	0.431	0.323	35.8	-
Platinum-based chemotherapy	12	1.49 (1.22–1.82)	0.000	0.000	73.6	0.146
Taxane-containing chemotherapy^b^	3	1.30 (0.91–1.85)	0.148	0.431	0.0	0.902
Non-taxane-containing chemotherapy^c^	5	1.91 (1.46–2.50)	0.000	0.000	88.6	0.110
All chemotherapy^d^	4	1.39 (0.97–1.99)	0.074	0.027	64.7	0.663
Geographical region						
America	1	0.95 (0.48–1.88)	0.883	-	-	-
Asia	3	2.04 (1.30–3.18)	0.002	0.907	0.0	0.119
Europe	6	1.27 (0.92–1.75)	0.141	0.000	87.6	-
Oceania	2	1.34 (1.10–1.62)	0.003	0.732	0.0	0.199
High ABCB1 expression adjusted for residual disease	4	2.13 (1.60–2.83)	0.000	0.000	85.4	0.003
*ABCB1* gene variants	4	0.95 (0.81–1,12)	0.555	0.003	78.4	0.582
TR	21					
High ABCB1 expression	21	0.63 (0.54–0.75)	0.000	0.000	66.6	0.019
ABCB1 detected by IHC	16	0.63 (0.52–0.75)	0.000	0.000	72.4	0.051
ABCB1 detected by by PCR	4	0.52 (0.33–0.80)	0.003	0.214	33.0	0.464
FIGO III-IV	13	0.57 (0.47–0.68)	0.000	0.000	69.9	0.010
Serous tumor	4	0.72 (0.57–0.91)	0.006	0.024	68.1	0.823
Platinum-based chemotherapy	21	0.63 (0.54–0.75)	0.000	0.000	63.7	0.019
Taxane-containing chemotherapy^b^	5	0.76 (0.49–1.16)	0.199	0.164	38.7	0.032
Non-taxane-containing chemotherapy^c^	13	0.59 (0.48–0.72)	0.000	0.000	73.2	0.032
All chemotherapy^d^	3	0.82 (0.53–1.28)	0.389	0.180	41.6	0.171
Geographical region						
America	2	0.85 (0.46–1.54)	0.587	0.024	80.3	-
Asia	9	0.62 (0.46–0.85)	0.003	0.271	19.4	0.133
Europe	10	0.62 (0.51–0.76)	0.000	0.000	77.2	0.547

a: HR and its 95%CI were used to assess OS and PFS, RR and its 95%CI were used to assess TR

b: PT (platinum + taxane) regimen

c: PAC (platinum + doxorubicin + cyclophosphamide), PA (platinum + doxorubicin) or PC (platinum + cyclophosphamide) regimen

d:Studies identified patients received PT, PAC, PA or PC regimen.

### Meta-analysis of the correlation between ABCB1 status and PFS in EOC

**Fig 5:**
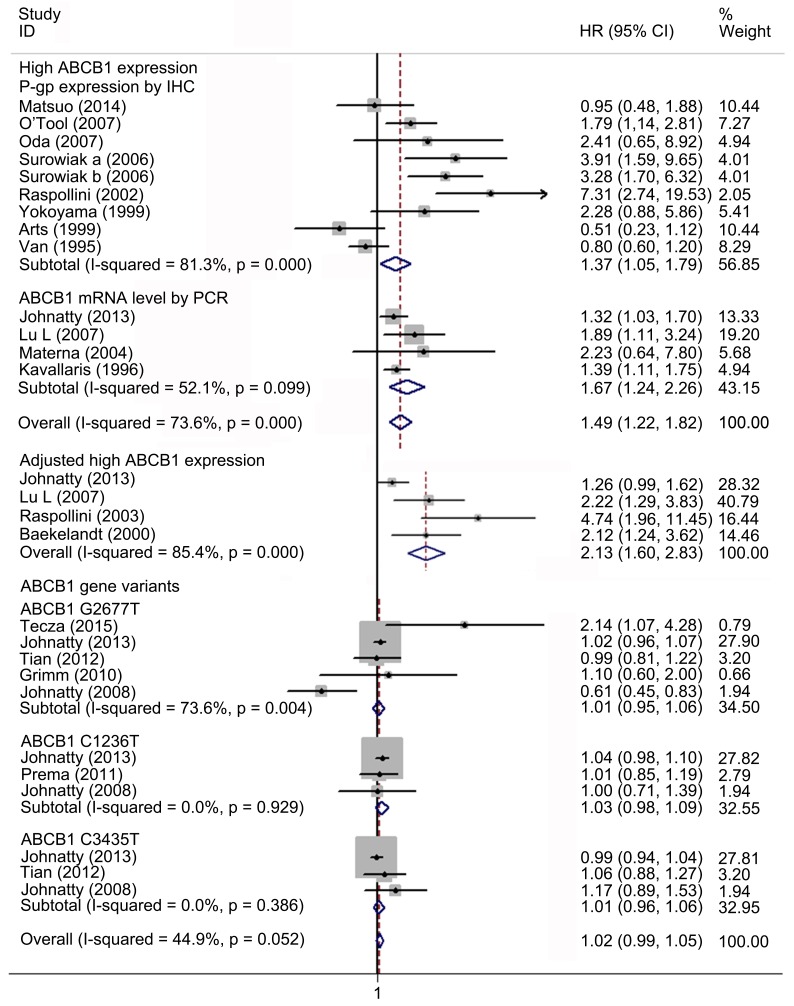
Forest plots presenting HRs of EOC PFS for high ABCB1 expression, adjusted results and *ABCB1* gene variants. HR = hazard ratio; EOC = epithelial ovarian cancer; PFS = progression free survival.

**Fig 6:**
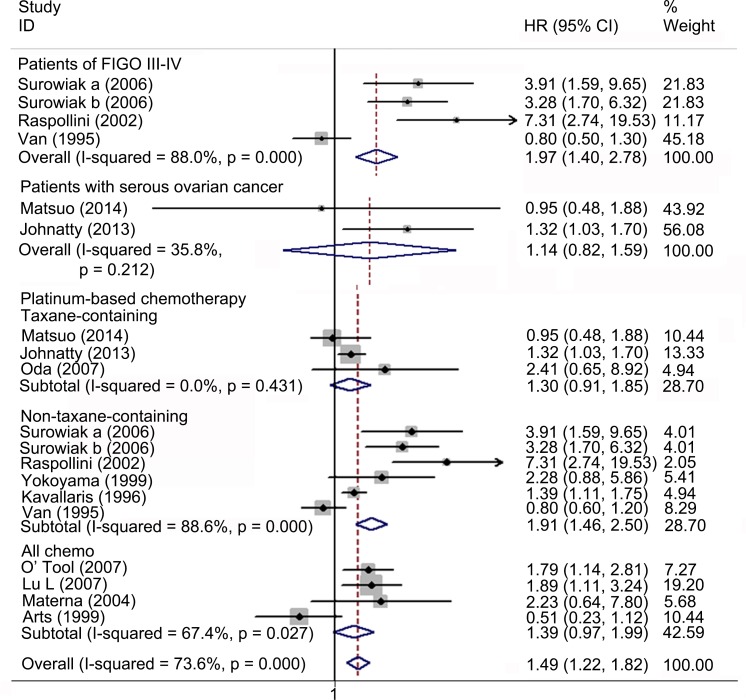
Forest plots of subgroup meta-analysis presenting HRs of EOC PFS for high ABCB1 expression. HR = hazard ratio; EOC = epithelial ovarian cancer; PFS = progression free survival.

**Fig 7:**
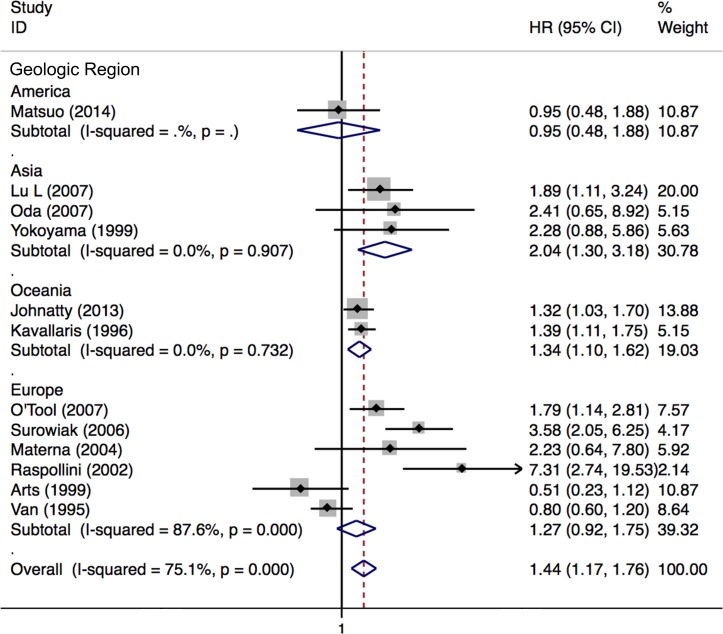
Forest plots of HRs of EOC PFS for high ABCB1 expression stratified by geographical region. HR = hazard ratio; EOC = epithelial ovarian cancer; PFS = progression free survival.

Nineteen studies with 7133 patients were included for meta-analysis of PFS. Fourteen studies with 1186 patients reported the association between high ABCB1 expression and PFS. Overall, high ABCB1 expression was associated with a poor PFS (HR 1.49; 95% CI: 1.22–1.82) in EOC. The effect of ABCB1 expression on PFS remained significant in the IHC (HR, 1.37; 95% CI: 1.05–1.79), the PCR (HR 1.67; 95% CI: 1.24–2.26), the FIGO III-IV (HR, 1.97; 95% CI: 1.40–2.78), the platinum-based non-taxane-containing chemotherapy (HR, 1.91; 95% CI: 1.46–2.50), the Asia (HR, 2.04; 95% CI: 1.30–3.18) and the Oceania (HR, 1.34; 95% CI: 1.10–1.62) subgroups. Similarly, when adjusted for residual disease (N = 4), age (N = 3), FIGO stage (N = 2), histological type (N = 1) and tumor grade (N = 1), high ABCB1 expression remained significantly associated with PFS (HR, 2.13; 95% CI: 1.60–2.83). However, such correlations were not found in the serous, the taxane-containing chemotherapy, the America and the Europe subgroups. Six studies with 5947 patients reported the association between rs2032582, rs1128503 or rs1045642 and PFS. No evidence of correlation between *ABCB1* gene SNPs and PFS was found (HR, 1.02; 95% CI: 0.99–1.05). Significant heterogeneities were found in overall meta-analysis of high ABCB1 expression (I^2^ = 73.6%, *P* = 0.000), the IHC (I^2^ = 81.3%, *P* = 0.000), the PCR (I^2^ = 52.1%, *P* = 0.099), the rs2032582 (I^2^ = 73.6%, *P* = 0.004), the FIGO stage (I^2^ = 88.0%, *P* = 0.000), the platinum-based (I^2^ = 88.6%, *P* = 0.000), the all chemo (I^2^ = 64.7%, *P* = 0.027), the Europe (I^2^ = 87.6%, *P* = 0.000) subgroups and when adjusted for residual disease (I^2^ = 85.4%, *P* = 0.000) (Figs 5–7 and Table 3).

### Meta-analysis of the correlation between ABCB1 status and TR in EOC

**Fig 8:**
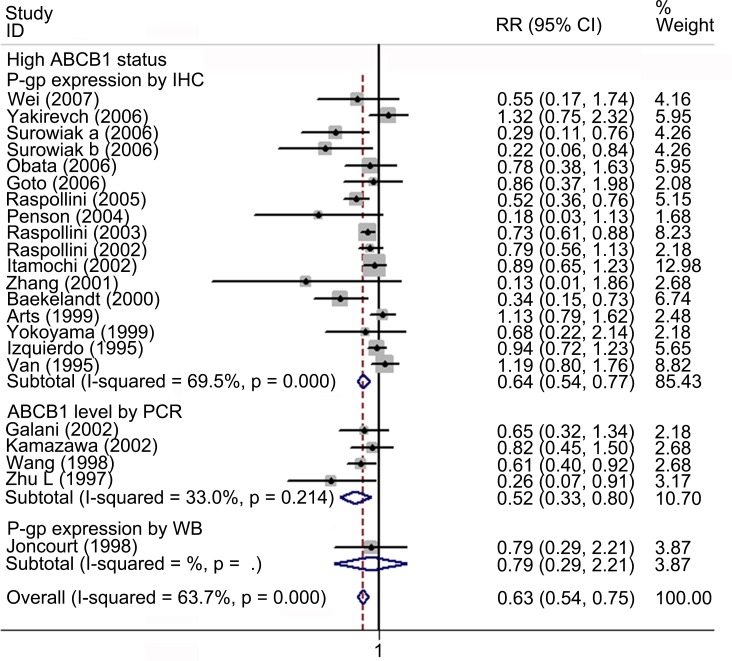
Forest plots presenting RRs of EOC TR for high ABCB1 expression. RR = risk ratio; EOC = epithelial ovarian cancer; TR = total response rate.

**Fig 9:**
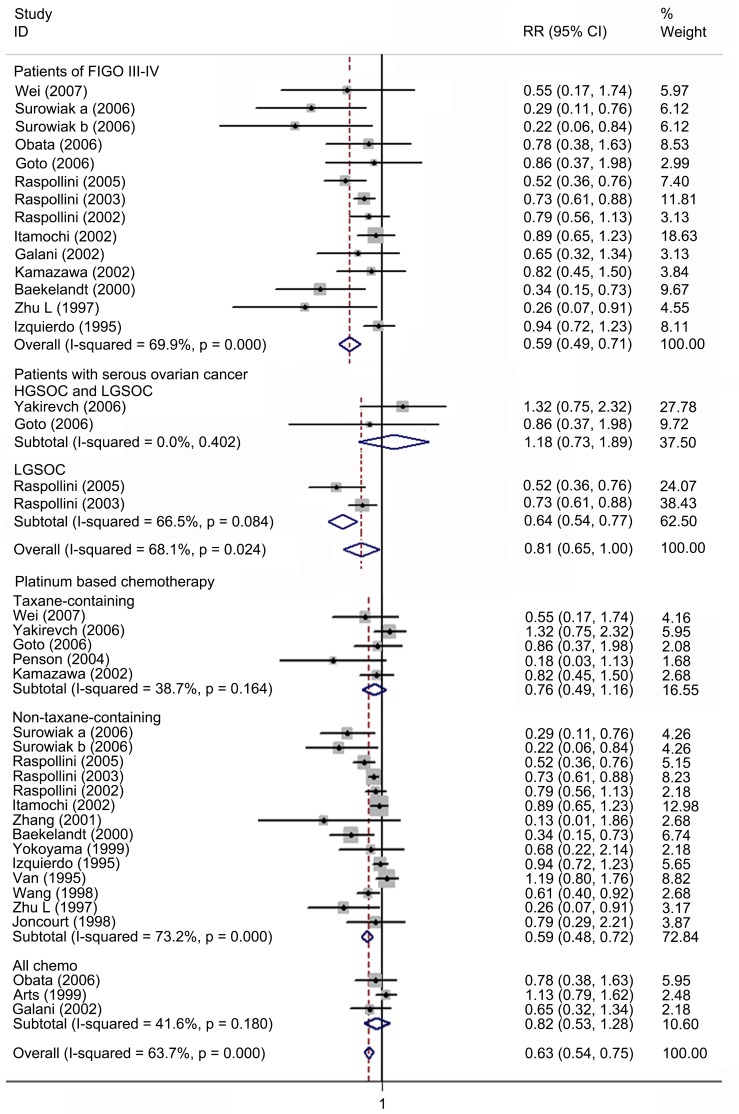
Forest plots of subgroup meta-analysis presenting RRs of EOC TR for high ABCB1 expression. RR = risk ratio; EOC = epithelial ovarian cancer; TR = total response rate.

**Fig 10:**
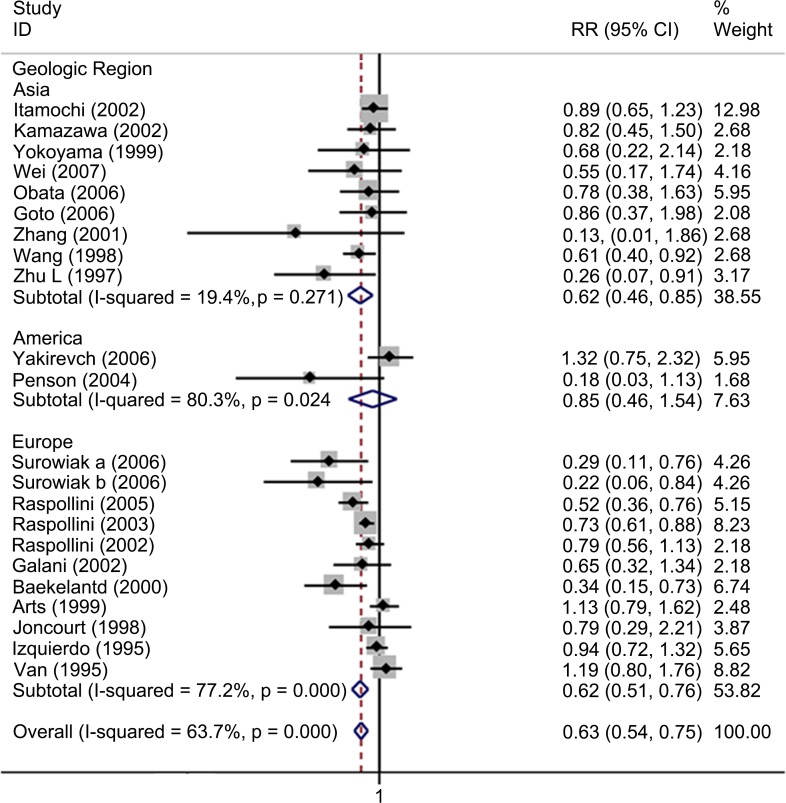
Forest plots of RRs of EOC TR for high ABCB1 expression stratified by geographical region. HR = hazard ratio; EOC = epithelial ovarian cancer; TR = total response rate.

Twenty-one studies involving 966 patients were included for meta-analysis of TR. Overall, high ABCB1 expression was associated with a poor TR (RR, 0.63; 95% CI: 0.54–0.75) in EOC. The effect of ABCB1 expression on OS remained significant in the IHC (RR, 0.63; 95% CI: 0.52–0.75), the PCR (RR, 0.52; 95% CI: 0.33–0.80), the FIGO III-IV (RR, 0.57; 95% CI: 0.47–0.68), the platinum-based non-taxane-containing chemotherapy (RR, 0.59; 95% CI: 0.48–0.72), the Asia (RR, 0.62; 95% CI: 0.46–0.85) and the Europe (RR, 0.62; 95% CI: 0.51–0.76) subgroups. No evidence for correlations between ABCB1 expression and TR were found in the serous, the taxane-containing and the all chemo subgroups. We found that in the serous subgroup, the correlation between ABCB1 expression and TR became significant in studies specifically identified low-grade serous ovarian cancer (LGSOC) and remained non-significant in studies identified HGSOC. The heterogeneities of overall meta-analysis (I^2^ = 66.6%, *P* = 0.000), the IHC (I^2^ = 72.4%, *P* = 0.000), the FIGO stage (I^2^ = 69.9%, *P* = 0.000), the serous (I^2^ = 68.1%, *P* = 0.024), the platinum-based (I^2^ = 73.2%, *P* = 0.000), the America (I^2^ = 80.3%, *P* = 0.024) and the Europe (I^2^ = 77.2%, *P* = 0.000) subgroups were significant (Figs 8–10 and Table 3).

### Publication Bias

**Fig 11:**
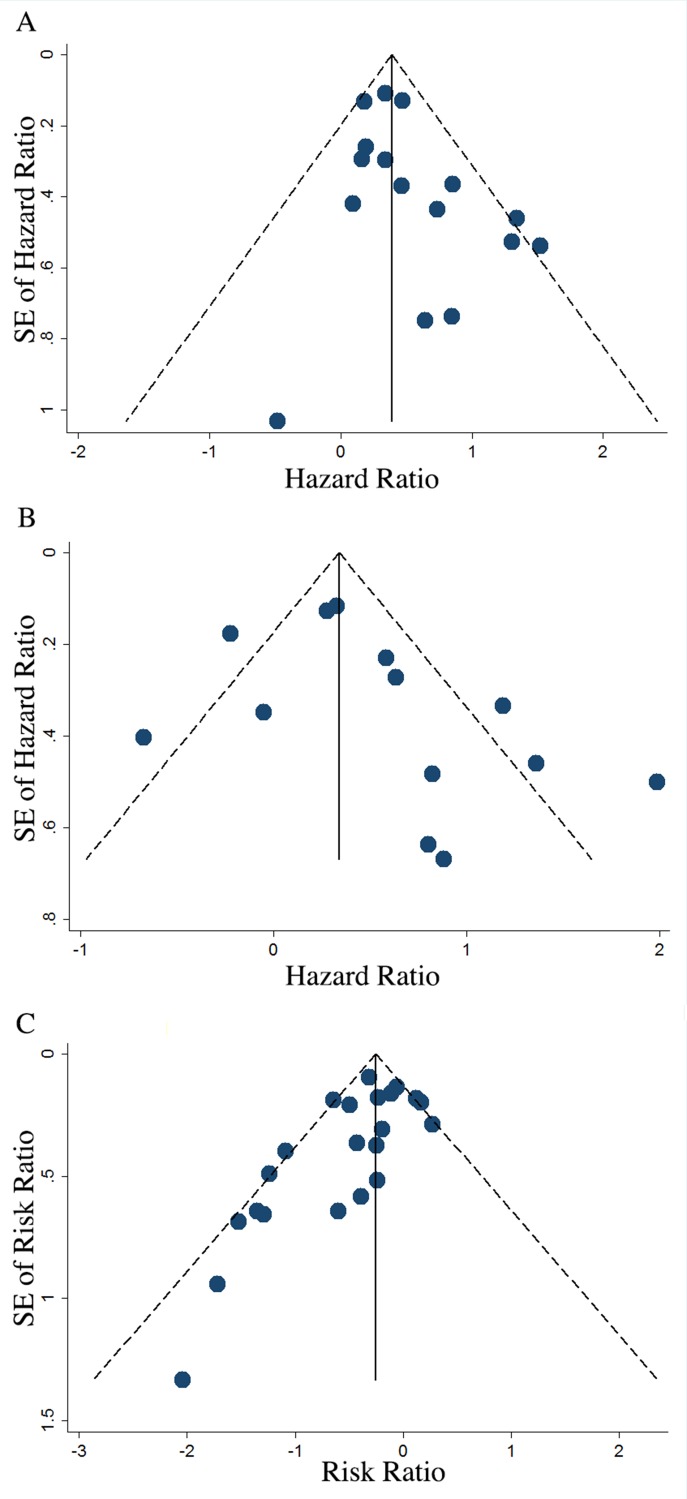
Funnel plots presenting prognostic value of ABCB1 for OS, PFS and TR. OS = overall survival; PFS = progression free survival; TR = total response rate. A, funnel plots showing the distribution of effect size and prevision of individual study estimate when evaluating the summary HR for OS. Egger’s test: *P* = 0.078. B, funnel plots showing the distribution of effect size and prevision of individual study estimate when evaluating the summary HR for PFS. Egger’s test: *P* = 0.146. C, funnel plots showing the distribution of effect size and prevision of individual study estimate when evaluating the summary RR for TR. Egger’s test: *P* = 0.019.

Publication bias was first assessed by visual inspection of funnel plots and then estimated with bias *p*-value from Egger’s test. While asymmetry of funnel plots was observed in overall meta-analysis of OS, PFS and TR, the results of egger’s test indicated that no publication bias existed in studies included for overall meta-analysis of OS, PFS and most subgroup meta-analysis. Publication bias was only found in studies for overall meta-analysis of TR (*P* = 0.019), analysis of PFS when adjusted for residual disease (*P* = 0.003), the FIGO stage (*P* = 0.010), the platinum-based (*P* = 0.032) and the taxane-containing (*P* = 0.032) subgroups of TR. After excluding certain studies to eliminate publication bias, the results of these analyses remained consistent to the original results (Fig 11 and Table 3).

## Discussion

Most EOC patients develop resistance to chemotherapy and eventually demise from the recurrent disease due to drug resistance. The development of chemo-resistance significantly impairs the survival in patients with EOC. The identification of predictive factors for chemo-resistance and poor survival might improve the individualized therapy for EOC. In the present study, we systematically assessed the impact of ABCB1, a transporter participating in efflux of a series of drugs, on chemotherapy response and survival in patients with EOC. We found that a high ABCB1 expression was significantly associated with decreased OS, PFS, and TR in EOC patients. These results are convincing because (i) the outcome definition was clear, (ii) the included studies were sub-grouped according to measuring methods to ensure the value of comparability, (iii) multivariate results further validated the association between ABCB1 and EOC prognosis and (iv) the number of studies reporting positive results or non-significant positive results were of greater quantity than the corresponding studies reporting negative results. Although heterogeneities were found in overall analyses for PFS and TR, we conducted subgroup analysis and determined histology and chemotherapy regimen as two main sources of heterogeneity between studies.

EOC consists of several histological subtypes, such as serous, mucinous, clear cell, endometrioid carcinomas. Different subtypes differ in genetic and biological features, clinical progression patterns, response to treatment, and prognosis. Therefore, it is meaningful to analyze the association between ABCB1 and prognosis in different ovarian cancer subtypes separately. However, in our subgroup analysis, we found only 6 out of 38 studies reported exclusively on serous ovarian carcinoma while the remaining reported on all subtypes as a whole. Since none of the studies reported independent data of any other subtypes other than serous ovarian carcinoma, we could not further determine what effects did other subtypes have on the significance association. Even though we find no evidence for correlation between ABCB1 expression and prognosis in patients with serous carcinoma, which accounts for 70% to 80% of all cases, the result is limited by small number of included studies (N = 6) therefore further validation is essential. Moreover, serous ovarian cancer can be classified into HGSOC and LGSOC according to the origins of tumorigensis, precursor lesions, genetic and molecular characteristics, morphologic features, clinical behaviors and chemotherapy responses [61,62]. HGSOC accounts for most ovarian cancer deaths as nearly 90% of the late stage HGSOC patients develop acquired resistance to platinum-based chemotherapy [63]. The mechanisms underlying acquired chemo-resistance in HGSOC include activation of AKT pathway, the reversion of BRCA1/BRCA2 germline mutations and ABCB1 overexpression [64]. Many argued that although decreased drug accumulation either by decreased influx, increased efflux or enhanced drug detoxification was identified as an important mechanism of drug resistance, most of these mechanisms were not clinically significant in HGSOC [65]. Similarly, we didn’t find any evidence for correlation between ABCB1 and prognosis of HGSOC patients. However, we did find unfavorable OS, PFS and TR accompanied by up-regulated ABCB1 in LGSOC patients, which is consistent with the fact that LGSOC is more resistant than HGSOC to initial chemotherapy (N = 2). Nevertheless, more studies were needed to validate the evolvement of ABCB1 in LGSOC chemo-resistance.

The regimen of platinum and taxane combination is the first-line treatment for EOC due to its superiority over other regimens regarding OS and adverse effects [66–68]. Although taxanes are substrates of ABCB1, our results did not reveal any evidence for a correlation between ABCB1 expression and prognosis in patients who received taxane-containing regimen. Instead, we found an adverse correlation between ABCB1 expression and prognosis in patients who received non-taxane-containing regimen, such as platinum combined with doxorubicin or cyclophosphamide. The biological effects resulted from up-regulated ABCB1 expression in the patients undergoing taxane-containing therapy might be compensated by other mechanisms that need further investigation. On one hand, it seemed that extensive use of taxanes successfully improved the general survival of EOC patients comparing to other drugs [69] and attenuated the sensitivity of ABCB1 as a prognosis indicator. On the other, since both cellular and clinical researches revealed that ABCB1 in ovarian cancer could be up-regulated by chemotherapy [70, 71], taxane induced alteration in ABCB1 through the course of treatment might also be a confounding factor to the measurement of survival analysis because ABCB1 expression was seldom obtained in matched analysis [72–74]. In pre-clinical studies, inhibitors targeting ABCB1 could reverse chemotherapy resistance to some extent [75,76]. However, the efficacies of these ABCB1 inhibitors were somehow discouraging in clinical trials for treatment of EOC patients with initial or acquired chemo-resistance [77]. Unselected patients might be one of the main reasons for that [78]. Our current results indicated that the patients undergoing non-taxane-containing chemotherapy rather than those undergoing taxane-containing chemotherapy might be benefited more from ABCB1 inhibition.

We observed an intimate relationship of ABCB1 expression in recurrent tumor with OS, but not that in primary tumor. Similarly, a whole-genome sequence analysis of a large cohort of sensitive, resistant and refractory ovarian carcinoma tissue specimens performed by Patch et al. revealed that ABCB1 was overexpressed in the recurrent and refractory tumors compared with the sensitive ones, which was caused by recurrent promoter fusion with SLC25A40 and led to acquired resistance to chemotherapy [79]. These findings suggest that the increase in ABCB1 induced by chemotherapy rather than the background ABCB1 expression is response for chemo-resistance and impaired survival and ABCB1 may play an essential role in the development of acquired drug resistance in ovarian cancer. However, the number of included studies for the ABCB1 in recurrent tumor was limited (*N* = 3) that restricts the reliability of this result.

To sum up, the present meta-analysis showed a significant negative correlation between ABCB1 expression level and prognosis in EOC patients and provided preliminary evidence highlighting this correlation in specific subgroups stratified by chemotherapy regimens, histological subtypes and tissues used for ABCB1 detection. Large-scale, prospective studies are needed to verify the clinical significance of ABCB1 expression in EOC.

## Supporting Information

S1 Checklist PRISMA checklist. (DOCX)

S2 Checklist Genetic associated meta-analysis checklist. (DOCX)

S1 File Quality assessment of the included studies. (DOCX)

S1 Table List of excluded articles and reasons for exclusion. (DOCX)
