# Supplementary material for: Prognostic Value and Implication for Chemotherapy Treatment of ABCB1 in Epithelial Ovarian Cancer: A Meta-Analysis
Source: PLoS One. 2016 Nov 3;11(11):e0166058. doi: 10.1371/journal.pone.0166058 (PMC5094734; doi:10.1371/journal.pone.0166058)
Supplement: S1 Table — (DOCX) [file pone.0166058.s004.docx]

**S1 Table. List of excluded articles and reasons for exclusion.**

| **Study** | **Reason** |
| --- | --- |
| Sedlakova I, Laco J, Caltova K, Cervinka M, Tosner J, Rezac A, et al. Clinical significance of the resistance proteins LRP, Pgp, MRP1, MRP3, and MRP5 in epithelial ovarian cancer. International journal of gynecological cancer : official journal of the International Gynecological Cancer Society. 2015;25(2):236-43. | Insufficient data |
| Lambrechts S, Lambrechts D, Despierre E, Van Nieuwenhuysen E, Smeets D, Debruyne PR, et al. Genetic variability in drug transport, metabolism or DNA repair affecting toxicity of chemotherapy in ovarian cancer. BMC pharmacology & toxicology. 2015;16:2. | Insufficient data |
| Sedlakova I, Laco J, Tosner J, Spacek J, Cermakova E. [Drug resistance proteins LRP, Pgp, MRP1, MRP3 and MRP5 in ovarian cancer patients]. Ceska gynekologie / Ceska lekarska spolecnost J Ev Purkyne. 2013;78(6):545-53. | Multiple publications |
| Brinkhuis M, Izquierdo MA, Baak JP, van Diest PJ, Kenemans P, Scheffer GL, et al. Expression of multidrug resistance-associated markers, their relation to quantitative pathologic tumour characteristics and prognosis in advanced ovarian cancer. Analytical cellular pathology : the journal of the European Society for Analytical Cellular Pathology. 2002;24(1):17-23. | Insufficient data |
| Katsaros D, Fracchioli S, Arts HJ, de Vries EG, Danese S, Richiardi G, et al. [Expression and prognostic value of the drug resistance markers P-gp, Mrp1, Mrp2, and Lrp in ovarian carcinoma]. Minerva ginecologica. 1999;51(12):463-70. | Insufficient data |
| Yin G, Chen S, Sun X. [Study on multidrug resistant gene (MDR1) expression between neoplastic cells and peripheral blood lymphocytes in ovarian carcinoma]. Zhonghua fu chan ke za zhi. 1998;33(5):287-9. | Insufficient data |
| Bourhis J, Goldstein LJ, Riou G, Pastan I, Gottesman MM, Benard J. Expression of a human multidrug resistance gene in ovarian carcinomas. Cancer research. 1989;49(18):5062-5. | Insufficient data |
| Peethambaram P, Fridley BL, Vierkant RA, Larson MC, Kalli KR, Elliott EA, et al. Polymorphisms in ABCB1 and ERCC2 associated with ovarian cancer outcome. International journal of molecular epidemiology and genetics. 2011;2(2):185-95. | Different outcome measures |
| Hamidovic A, Hahn K, Kolesar J. Clinical significance of ABCB1 genotyping in oncology. Journal of oncology pharmacy practice : official publication of the International Society of Oncology Pharmacy Practitioners. 2010;16(1):39-44. | Review |
| Green H, Soderkvist P, Rosenberg P, Horvath G, Peterson C. ABCB1 G1199A polymorphism and ovarian cancer response to paclitaxel. Journal of pharmaceutical sciences. 2008;97(6):2045-8. | Different outcome measures |
| Ren L, Xiao L, Hu J. MDR1 and MDR3 genes and drug resistance to cisplatin of ovarian cancer cells. Journal of Huazhong University of Science and Technology Medical sciences = Hua zhong ke ji da xue xue bao Yi xue Ying De wen ban = Huazhong keji daxue xuebao Yixue Yingdewen ban. 2007;27(6):721-4. | Original article |
| Marsh S, Paul J, King CR, Gifford G, McLeod HL, Brown R. Pharmacogenetic assessment of toxicity and outcome after platinum plus taxane chemotherapy in ovarian cancer: the Scottish Randomised Trial in Ovarian Cancer. Journal of clinical oncology : official journal of the American Society of Clinical Oncology. 2007;25(29):4528-35. | Insufficient data |
| Marsh S, King CR, McLeod HL, Paul J, Gifford G, Brown R. ABCB1 2677G>T/A genotype and paclitaxel pharmacogenetics in ovarian cancer. Clinical cancer research : an official journal of the American Association for Cancer Research. 2006;12(13):4127; author reply -9. | Insufficient data |
| Green H, Soderkvist P, Rosenberg P, Horvath G, Peterson C. mdr-1 single nucleotide polymorphisms in ovarian cancer tissue: G2677T/A correlates with response to paclitaxel chemotherapy. Clinical cancer research : an official journal of the American Association for Cancer Research. 2006;12(3 Pt 1):854-9. | Different outcome measures |
| Huang X, Ushijima K, Komai K, Takemoto Y, Motoshima S, Kamura T, et al. Co-expression of Y box-binding protein-1 and P-glycoprotein as a prognostic marker for survival in epithelial ovarian cancer. Gynecologic oncology. 2004;93(2):287-91. | Insufficient data |
| Schneider J, Jimenez E, Marenbach K, Marx D, Meden H. Co-expression of the MDR1 gene and HSP27 in human ovarian cancer. Anticancer research. 1998;18(4C):2967-71. | Insufficient data |
| Surowiak P, Materna V, Kaplenko I, Spaczynski M, Dolinska-Krajewska B, Gebarowska E, et al. ABCC2 (MRP2, cMOAT) can be localized in the nuclear membrane of ovarian carcinomas and correlates with resistance to cisplatin and clinical outcome. Clinical cancer research : an official journal of the American Association for Cancer Research. 2006;12(23):7149-58. | Insufficient data |
| Guminski AD, Balleine RL, Chiew YE, Webster LR, Tapner M, Farrell GC, et al. MRP2 (ABCC2) and cisplatin sensitivity in hepatocytes and human ovarian carcinoma. Gynecologic oncology. 2006;100(2):239-46. | Different outcome measures |
| Auner V, Sehouli J, Oskay-Oezcelik G, Horvat R, Speiser P, Zeillinger R. ABC transporter gene expression in benign and malignant ovarian tissue. Gynecologic oncology. 2010;117(2):198-201. | Different outcome measures |
| Obata H, Yahata T, Quan J, Sekine M, Tanaka K. Association between single nucleotide polymorphisms of drug resistance-associated genes and response to chemotherapy in advanced ovarian cancer. Anticancer research. 2006;26(3B):2227-32. | Insufficient data |
